# Injectable Hydrogel for Synergetic Low Dose Radiotherapy, Chemodynamic Therapy and Photothermal Therapy

**DOI:** 10.3389/fbioe.2021.757428

**Published:** 2021-11-22

**Authors:** Mingzhu Chen, Ziqi Wang, Weilong Suo, Zhirong Bao, Hong Quan

**Affiliations:** ^1^ Key Laboratory of Artificial Micro- and Nano-Structures of Ministry of Education, School of Physics and Technology, Wuhan University, Wuhan, China; ^2^ Key Laboratory of Applied Chemistry and Nanotechnology at Universities of Jilin Province, Changchun University of Science and Technology, Changchun, China; ^3^ Hubei Key Laboratory of Tumor Biological Behaviors, Department of Radiation and Medical Oncology, Hubei Cancer Clinical Study Center, Zhongnan Hospital of Wuhan University, Wuhan, China

**Keywords:** FeGa, fenton reaction, radiotherapy, hydrogel, tumor therapy

## Abstract

Higher doses of radiotherapy (RT) are associated with resistance induction, therefore highly selective and controllable radiosensitizers are urgently needed. To address this issue, we developed a FeGA-based injectable hydrogel system (FH) that can be used in combination with low-dose radiation. Our FH can deliver FeGA directly to the tumor site via intratumoral injection, where it is a reservoir-based system to conserve FeGA. The photothermal properties of FeGA steadily dissolve FH under laser irradiation, and, simultaneously, FeGA reacts with a large amount of H_2_O_2_ in the cell to produce OH (Fenton reaction) which is highly toxic to mitochondria, rendering the cell inactive and reducing radiotherapy resistance. *In vivo* and *in vitro* studies suggest that combining the FH and NIR irradiation with RT (2Gy) can significantly reduce tumor proliferation without side effects such as inflammation. To conclude, this is the first study to achieve combined chemodynamic therapy (CDT) and photothermal therapy (PTT) *in situ* treatment, and the best therapeutic effect can be obtained with a low-dose radiation combination, thus expanding the prospects of FeGA-based tumor therapy.

## Introduction

Cancer, which can strike at any age and affect anybody, is a serious threat to human life and health in society of today despite current scientific advancements (Agostinis et al., 2011; Bonfiglio et al., 2017). Radiotherapy (RT) is utilized to keep over half of all cancer patients alive, either alone or in combination with other cutting-edge treatments (Kwatra et al., 2013; Huang et al., 2021). RT is based on the use of high-energy X-rays or gamma rays to generate radiation-induced DNA damage or to trigger the development of huge amounts of harmful reactive oxygen species (ROS), of which mitochondria produce around 90% (Cavalli et al., 2016; Park et al., 2017). Mitochondria are the most important organelles in radiosensitization. When the rate of ROS production exceeds the potential of the cell to neutralize these free radicals, cell death occurs. This kills tumor cells and reduces the size of the tumor. However, while RT kills tumor cells, it does so at the expense of nearby cells and tissues in the human body (Xie et al., 2019; Liu et al., 2020). High-dose radiation rays can produce more ROS that attacks tumor DNA, resulting in better treatment outcomes (Tennant et al., 2010). High-dose radiotherapy, on the other hand, might cause systemic effects as well as local radiation damage. Fatigue, loss of appetite, bone marrow suppression, and radiotherapy-induced secondary primary malignancies and infertility are all common systemic responses (Spencer et al., 2018). Local liver injury can lead to altered or even liver failure in more serious cases (Escorcia et al., 2007). The tumor microenvironment, in particular, includes a lot of H_2_O_2_ (Li et al., 2020). If alternative catalysts can be employed to produce •OH to damage mitochondria, it is expected to have a good synergistic impact when paired with irradiation, and the dose of radiation will be reduced without compromising the therapeutic effect.

Based on this, researchers have designed a range of different treatments to raise the tumor hydroxyl radicals (•OH) levels. More and more research studies suggest that using exogenous iron to treat CDT has significant benefits (Ni et al., 2020; Alizadeh and Salimi, 2021). In the tumor microenvironment, Fe^2+^ can react with a large amount of H_2_O_2_ to produce toxic •OH *in situ* and damage mitochondria (Ranji-Burachaloo et al., 2018). Several iron-containing formulations, including ferroferric oxide nanoparticles, iron oxide nanoparticles, and FeGA particles, have been used alone or in combination with other types of treatments to act as Fenton reaction catalysts to induce cell death (Dong et al., 2019; Chen D. et al., 2020; Yang et al., 2021). Gallic acid and Fe^2+^ were employed by Liu et al. to prepare ultra-small FeGA complexes that can achieve CDT indefinitely. Simultaneously, GA-mediated Fe^3+^ to Fe^2+^ conversion improved the catalytic stability of free Fe^2+^ when combined with the glutathione (GSH) depletion agent L-buthionine sulfoximine (BSO) (Dong et al., 2019). Thus, improved tumor oxidative stress was achieved that considerably boosted the therapeutic efficacy of concurrent chemotherapy or radiotherapy. Although their experiments has achieved a certain anti-tumor effect. Intravenous injection and blood circulation deliver these nanoparticles to tumor tissues. Because of poor immune escape capacity and rapid elimination of these nanoparticles from the bloodstream by the action of liver and kidney organs, their transfer effect and anti-tumor efficacy are significantly diminished (Zheng et al., 2018; Yin et al., 2019; Chen G. et al., 2020). Although liposomes and other drug delivery systems have achieved greater efficacy in tumor treatment in recent years, drug delivery systems also face interference and a series of biological barrier effects, such as the clearance of liver and kidney systems, cell resistance to foreign drugs and so on (Yong et al., 2019; Yang et al., 2020; Wang et al., 2021; Zhang et al., 2021).

Traditional drug delivery systems are susceptible to issues such as poor drug loading, complicated synthesis methods, early drug leakage or slow release, and the long-term toxicity brought on by the carrier’s presence in the body over an extended period. As a controlled drug release platform, light-responsive hydrogels with minimum invasiveness have recently received a lot of attention (Qiu et al., 2018; Meng et al., 2019; Chen Q. W. et al., 2020). The hydrogel progressively solidifies after being injected into tumor tissue and can be utilized as a nanomaterial controlled release for a long period. After one injection, this form of local administration can be used repeatedly. Furthermore, parameters such as power and laser irradiation period can be modified to alter the medication release rate, extending the treatment method’s applicability (Zhu et al., 2021). For the first time, Zhu et al. employed an agarose hydrogel to deliver anti-tumor aggregation-induced emission materials (AIEgen) material. As a photothermal agent, Prussian blue (PB) nanozyme can stimulate the disintegration of the hydrogel while also acting as a catalase (CAT) enzyme to catalyze H_2_O_2_ to improve the tumor microenvironment (Zhu et al., 2021). Following that, under the irradiation of low-power white light, AIEgens can produce ROS under sufficient oxygen levels to promote tumor ablation. Zhang et al. developed a black phosphorus-based injectable hydrogel for photothermal therapy (Qiu et al., 2018), which employs external photoexcitation to release cancer medicines, resulting in accurate and safe cancer treatment. As a result of these findings, we believe that using hydrogels to deliver FeGA to damage mitochondria and improve the efficacy of low-dose radiation will be beneficial.

**SCHEME 1:**
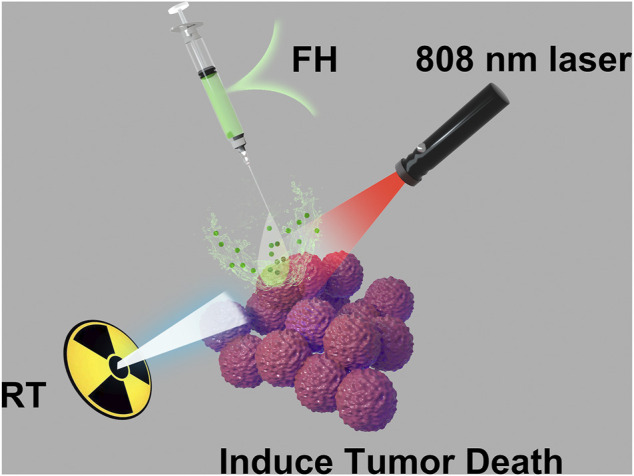
Injectable hydrogel for synergetic low dose radiotherapy, chemodynamic therapy and photothermal therapy.

In this study, we developed an injectable hydrogel containing FeGA nanoparticles for intratumoral delivery of photothermal and chemodynamic therapy, as well as radiation sensitization (Scheme 1). The United States Food and Drug Administration (FDA) has approved FeGA and agarose hydrogels owing to their reliable biosafety. To prepare the FeGA reservoir and release controller system (FH), the FeGA nanoparticles were loaded into an agarose hydrogel. FeGA nanoparticles serve as a photothermal agent (PTA) in this system due to their outstanding photothermal performance. FeGA turns light energy into heat energy when an 808 nm near-infrared (NIR) laser irradiates the FH system, causing the temperature of the agarose hydrogel to rise and reversible melting and softening to occur. FeGA diffuses into the local tumor microenvironment, and the Fenton reaction catalyzed by Fe^2+^ can produce a high amount of •OH, which can damage tumor cell mitochondria and increase radiation sensitivity. The FH can be employed as a FeGA storage controller to achieve regulated drug release and for intratumor injection of local tumor. This is the first report to achieve the combined treatment of CDT and PTT *in situ* and that the highest therapeutic result can be obtained when low-dose radiation was used in combination. In conclusion, the FH nanosystem has a wide range of clinical potential in synergetic therapy.

## Characterization of the FH

**FIGURE 1:**
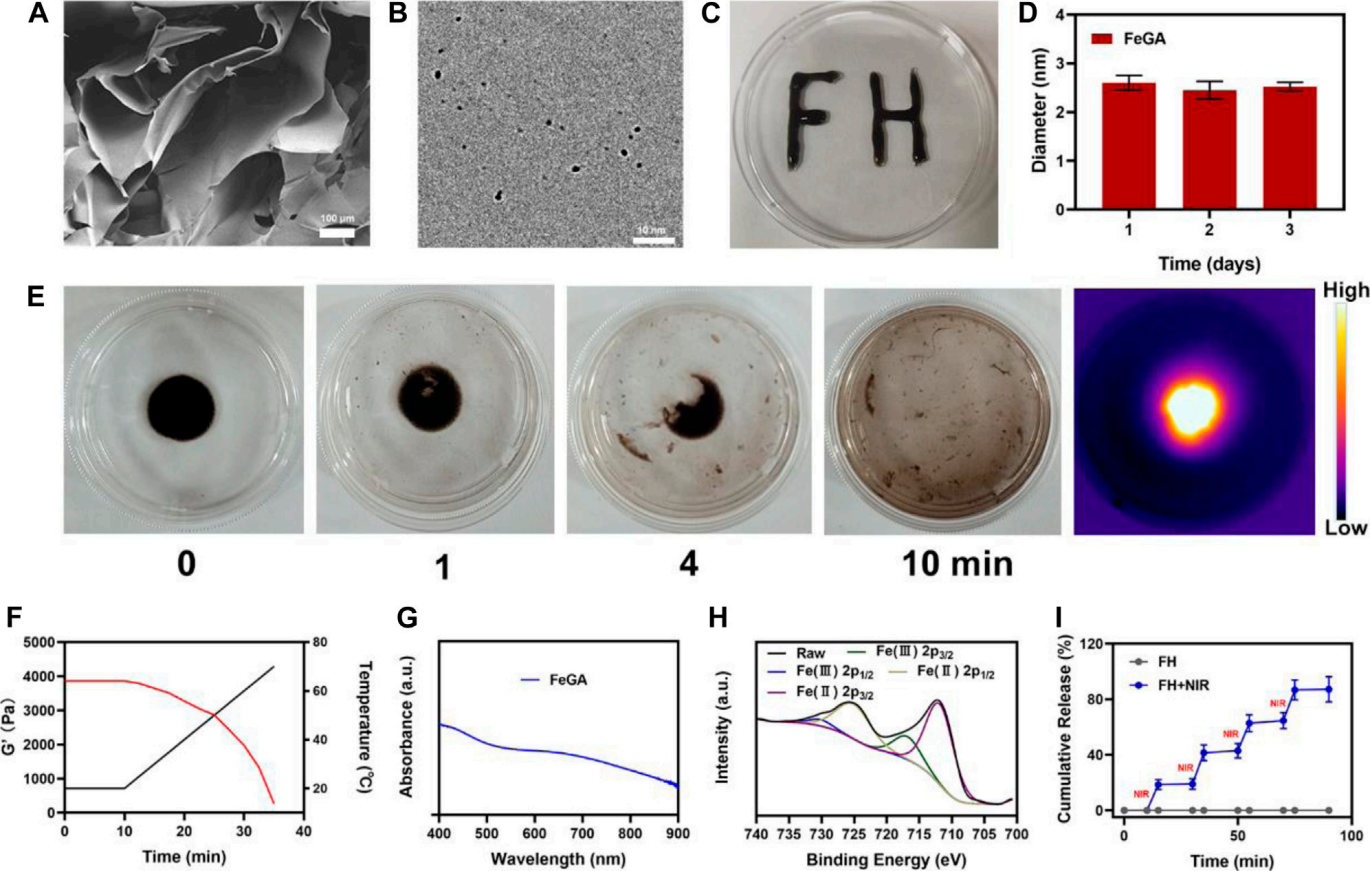
**(A)** SEM image of hydrogel. **(B)** TEM image of FeGA. **(C)** Written using the prepared FH via syringe, demonstrating the stability of this compound. **(D)** Statistical graph of measured diameter size of FeGA. **(E)** The morphology of the prepared FH before and after 0.5 W/cm^2^ 808 nm laser irradiation for 10 min and infrared thermal images of the prepared FH following irradiation. **(F)** Rheological and temperature curves (red and black, respectively) for the prepared FH under conditions that simulate an exposure to 0.5 W/cm^2^ 808 nm laser irradiation. **(G)** FeGA absorbance spectra. **(H)** XPS spectra of Fe 2p orbit in FeGA. **(I)** *In vitro* FeGA release profile in the presence and absence of 808 nm laser irradiation, with NIR being used to indicate irradiation time points.

To begin, the hydrogel was prepared using a basic hydrothermal technique (Figure 1A). The SEM images reveal the hydrogel’s complex pore structure. Figure 1B displayed the transmission electron microscope (TEM) image of FeGA. According to the results, FeGA has small size with an average of 2.52 ± 1.6 nm. Because nanomaterials smaller than 10 nm are easily cleared by the kidney, thus it is difficult to achieve the desired therapeutic effect (Zhou et al., 2018; Luo et al., 2016). As a result, the hydrogel delivery technique will considerably improve the applicability of FeGA. Moreover, when the particle size of FeGA was measured for three consecutive days, the size fluctuation range was very small (Figure 1D), indicating that FeGA has good stability, making it a good clinical application prospect. Although many materials have beneficial biological effects, their extremely low stability limits their long-term value. After solidification, the prepared composite hydrogel FH is very stable after being solidified (Figures 1C,E) demonstrates FH’s excellent photothermal heating properties. The prepared hydrogel is still solidified. After 10 min of 808 nm laser irradiation, the hydrogel was almost completely dissolved and FeGA nanomaterials were released. Infrared thermal imaging also confirmed that after irradiation, the FH temperature increased significantly. FH’s rheological values were measured at various temperatures (Figure 1F), and the results revealed that as the temperature rises, FH gradually dissolves, accompanied by a gradual decrease in storage modulus. This is in line with the hydrogel’s rheological properties (Hou et al., 2018). Figure 1G depicts FeGA’s UV-visible absorption. FeGA has a broad absorption spectrum in the 600–800 nm range, with a distinctive peak near 600 nm. X-ray photoelectron spectroscopy (XPS) was used to determine the Fe 2p spectrum in FeGA (Figure 1H), the peaks with binding energies of 725.8 and 712.1 eV corresponded to Fe (II) 2p_1/2_ and Fe (II) 2p_3/2_, respectively. Following that, we evaluated the FH controlled release material’s capability and the results are depicted in (Figure 1H). Laser irradiation partially dissolved FH, thus releasing FeGA. The hydrogel cooled and hardened after the laser irradiation was discontinued, the drug continues to be held in hydrogels. The drug was almost completely released after four laser switching cycles, demonstrating that our FH system has a powerful ability to release pharmaceuticals by controlled blasting.

## Mitochondria Damage Assessment *In vitro*

**FIGURE 2:**
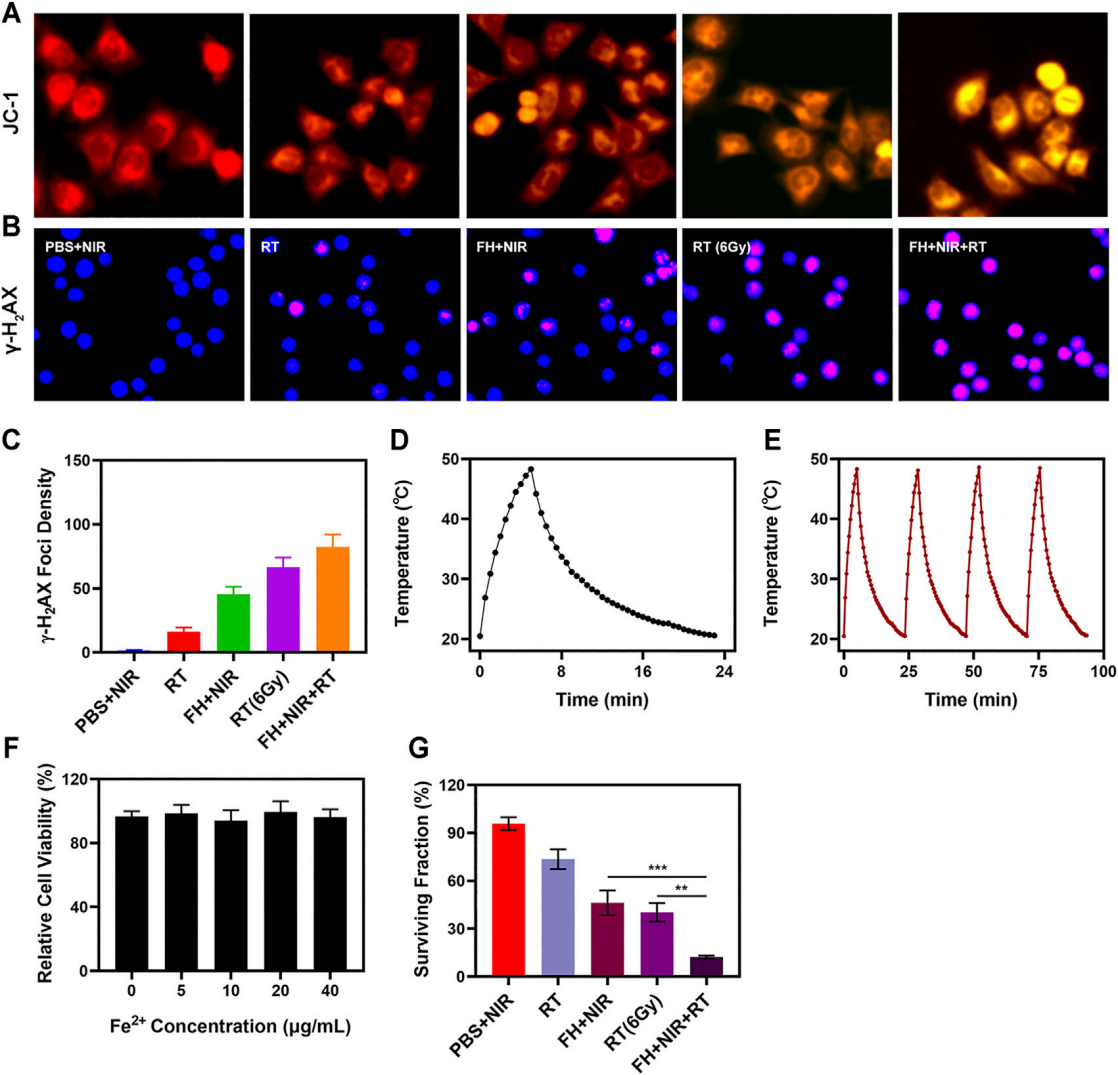
**(A)** JC-1 (green for JC-1 monomer and red for JC-1 aggregate fluorescence image under different treatment. **(B)** DAPI and *γ*-H2AX staining were used to respectively visualize nuclear condensation and DNA fragmentation in cells treated as indicated, with representative images being shown. **(C)** The density of *γ*-H2AX foci was determined based on analyses of 100 cells per treatment group (*γ*-H2AX foci/100 μm^2^, *n* = 3). **(D)** Temperature profile of a FeGA solution at 200 μg/ml upon heating when the laser is on and subsequent cooling once the laser is turned off. **(E)** Temperature variations of a FeGA solution over four cycles of heating and natural cooling. **(F)** Dark cytotoxicity of FeGA on 4T1 cells. **(G)** Colony formation assays were conducted using 4T1 cells treated with radiation (*n* = 3). **p < 0.01, ***p < 0.005; Student’s *t*-test.

The FH system is well-structured and performance-oriented. Anti-tumor experiments *in vitro* are presently underway. While FeGA has the potential to disrupt the ecological balance of cancer cells and hence increase the efficacy of radiation, it can do so only when it is present in tumor tissue. The immune system may recognize stimuli from a variety of foreign invaders, and a portion of that stimulation may trigger the immune response, resulting in immunity, while other stimuli may result in tolerance (Hu et al., 2016; Douglas, 2003). Hydrogels can be used to deliver drugs directly to tumor cells, effectively bypassing immune reactions (Zhu et al., 2021). The changes of mitochondrial membrane potential (MMP) in tumor cells were monitored by JC-1 (5,5′,6,6′-tetrachloro-1,1′,3,3′-tetraethyl-imidacarbocyanine) probe method. Normally, the JC dye accumulates in mitochondria, where it clumps together to produce red fluorescence. However, when mitochondrial membranes are damaged and MMP levels are low, the JC monomer is released into the cytoplasm, resulting in green fluorescence (Li et al., 2018). As illustrated in Figure 2A, cells treated with a low dose of RT (2Gy) in combination with laser irradiation and FH showed a high green/red fluorescence ratio, consistent with the enhanced mitochondrial damage generated by FeGA. FeGA would catalyze enough H_2_O_2_ to create •OH *in situ* once released by FH, causing substantial mitochondrial damage. Furthermore, as shown in Supplementary Figure S1, The control group and individual NIR showed dark fluorescence, indicating a relatively low concentration of OH. NIR + FH treatment group showed the strongest green fluorescence, because FeGA can catalyze endogenous H_2_O_2_ to produce OH in TME. As a result of mitochondrial damage, cells are more sensitive to radiotherapy. When tumor cells are exposed to radiation, double-stranded DNA breaks (DSB) occur, providing insight into radiation sensitization (Meng et al., 2018). Measuring the fluorescence intensity of γ -H_2_AX (a marker of DNA damage response) is a good and sensible technique to validate DSB formation following cell damage. Therefore, after treatment in several groups, we found H_2_AX foci in the nucleus. There was substantial DNA damage after 2 Gy of radiation, and when the dose was raised, the DSB effect increased. It is important to mention that 808 nm laser irradiation combined with FH only achieved about 45.6% γ-H2AX formation, notably; FH + NIR + RT got up to 82.4% γ-H_2_AX foci development.

## Photo-Thermal Properties of the FH

The uniform and significant difference between each experimental group were linked to the synergistic effect of FeGA as a photothermal agent in PTT, Fenton reaction to produce •OH to kill mitochondria and radiation sensitization. One of the most essential factors for evaluating PTA is photothermal stability. The 200 μg/ml FeGA solution was repeatedly heated with the 808 nm NIR laser for 5 min to assess the photothermal stability of FeGA nanoparticles (Figures 2D,E), then the solution was allowed to cool to ambient temperature. The heating curves of each cycle were identical, and the differences in peak temperature changes were minor, demonstrating that the FeGA nanoparticle’s photothermal conversion ability was found to be stable and repeatable over repeated four cycles of heating and cooling. These findings suggest that the FeGA nanospheres have good photothermal stability.

## *In vitro* Combination Therapy

FeGA was added to 4T1 cells at various doses (0, 5, 10, 20, 40 μg/ml) and incubated for 24 h. Even at high concentrations, cell viability did not decline significantly. FeGA NPs exhibit high biocompatibility, according to the findings. Above all, FeGA nanoparticles were found to be an excellent PTA for photothermal anti-tumor conversion. Colony formation assays also showed that the NIR with the FH group demonstrated considerable tumor growth inhibition (Figure 2H). FH + NIR + RT system had the best tumor growth inhibition rate (90%), there are significant differences compared with each other experiment group, indicating that FH-mediated improved concentration of OH of tumor microenvironment (TME) can exert influence on mitochondria and thus enhance the RT effect to realize tumor cells growth inhibition. These findings, taken together, motivate us to fully investigate anti-tumor efficacy *in vivo*.

## *In vivo* Anti-Tumor Experiments

**FIGURE 3:**
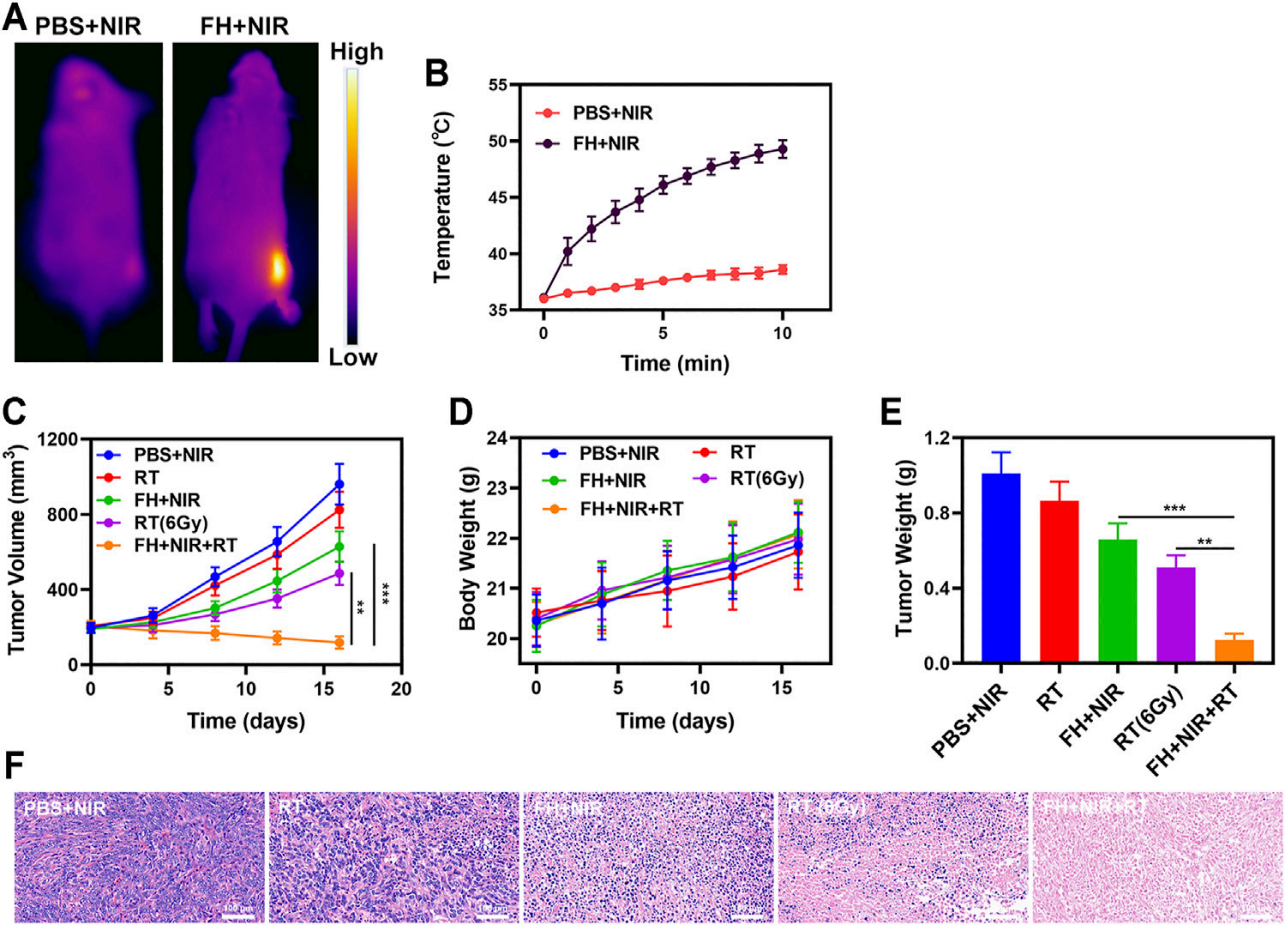
**(A)** IR thermal images of tumors following an 808 nm laser irradiation (0.5 W/cm^2^) for 5 min in the indicated treatment groups. **(B)** Temperature increases in mice implanted with 4T1 tumors following 808 nm laser irradiation (0.5 W/cm^2^) for 5 min in the indicated treatment groups. **(C)** Tumor volume change over time in groups treated as indicated. **(D)** Changes in body weight in response to the indicated treatments. **(E)** Average tumor weight values associated with the indicated treatments. **(F)** H and E stained tumor sections from the indicated treatment groups. **p < 0.01, ***p < 0.005; Student’s *t*-test.

We continued to investigate the photothermal conversion impact of FeGA *in vivo* due to its good *in vitro* performance as a PTA and radiosensitizer. BALB/c mice were used to develop 4T1 subcutaneous tumor models. Figures 3A,B show infrared pictures captured by a thermal imager, as well as temperature change curves for the PBS and FH groups after 10 min of 808 nm NIR laser irradiation at 0.5 W/cm^2^. Within 10 min after receiving FH, the temperature raised from 36.1°C to 49.3°C, whereas the PBS group experienced a little increase in temperature. Tumor tissues had lower heat resistance than normal cells, resulting in tumor cells being selectively destroyed at high temperatures (42–47°C) (Ouyang et al., 2019). The efficacy of FH-mediated anti-tumor activity was then tested in mice with 4T1 tumors. To investigate the primary effect of the FH, BALB/c mice were subcutaneous injected with 1 × 106 4T1 cells into the right flank. When the primary tumor volume reached 200 mm^3^, the mice were divided into groups and treated. Tumor-bearing mice were divided randomly into 5 groups (each group included 5 mice): 1) PBS + NIR; 2) Radiotherapy (RT, 2Gy); 3) FH + NIR; 4) High dose RT (6Gy); 5) PBS + NIR + RT. The FeGA concentration was 5 mg/kg in groups 3, and 5. For 16 days, the therapy was given every 4 days. The tumor volumes of the PBS + NIR group and the low dosage RT treated group increased fast over the 2 weeks of treatment, as illustrated in Figure 3C. The FH + NIR group also had a tumor-suppressing impact that was almost moderate. Once subjected to laser radiation after intratumoral injection of FH, it will hopefully release the FeGA particles out of the FH hydrogel. FeGA reacts with intratumoral H_2_O_2_ to produce •OH *in situ*, which destroys mitochondria and increases radiation sensitization. The FH + NIR + RT system, which included both FeGA, had the most potent therapeutic impact, with growth curves of tumor volume nearly totally suppressed during therapy. The tumor mass of mice was also in agreement with the volume curve (Figure 3E). No weight changes were observed in the treatment group throughout the study, indicating that the treatment did not cause any significant systemic toxicity (Figure 3D), which is noteworthy because many treatments are associated with severe systemic toxicity, which is incredibly harmful to the material’s future medical applications (Aminabee et al., 2020). We obtained slices of tumor tissue for staining. H&E staining (Figure 3F) revealed that the FH + NIR + RT group had a significant percentage of cell necrosis.

## *In vivo* Toxicity

**FIGURE 4:**
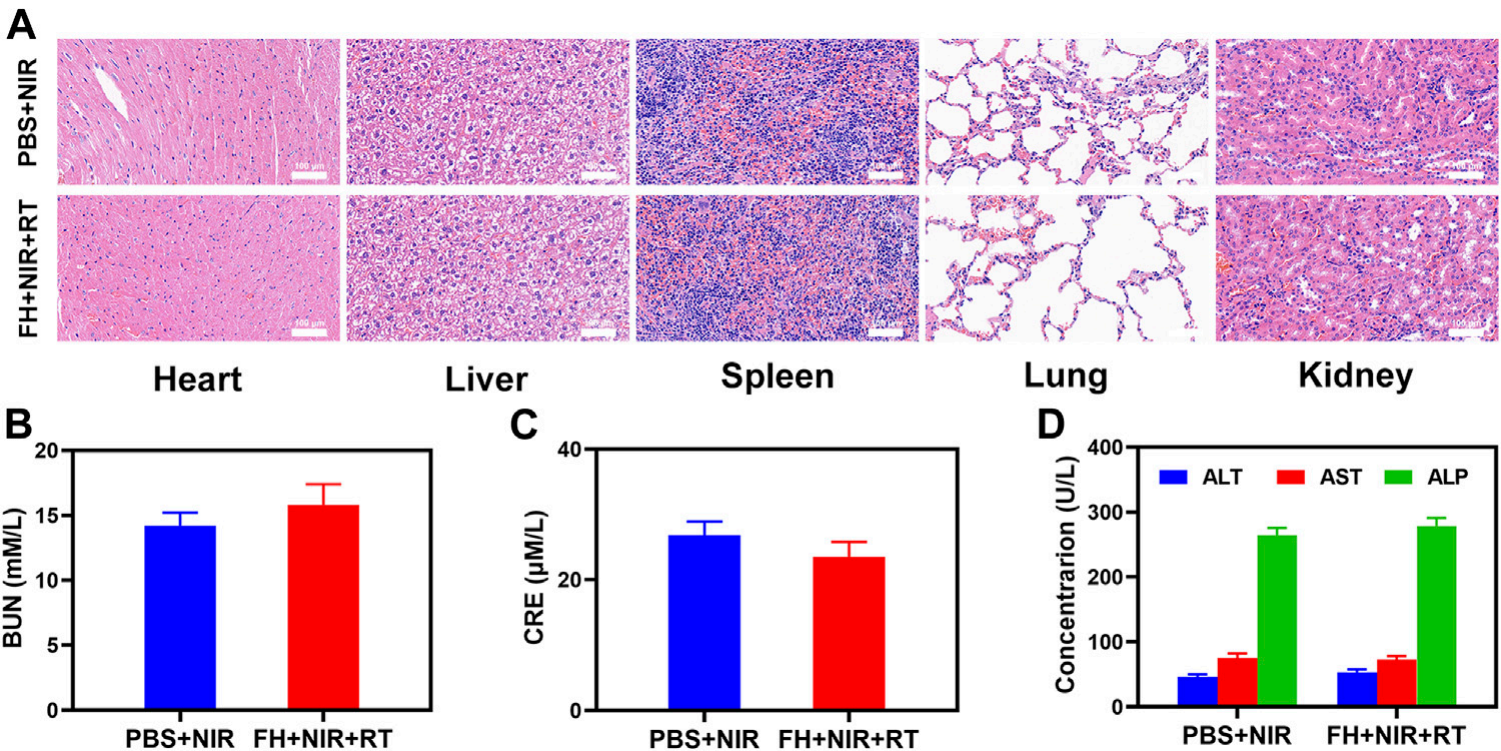
Result of *in vivo* safety experiments. **(A)** Histopathological analysis results (H and E stained images) of the major organs, heart, lung, liver, kidneys, and spleen, of mice that were exposed to different treatments 16 days post-injection. Blood biochemistry data including kidney function markers: **(B)** liver function markers: BUN, **(C)** CRE, and **(D)** ALT, ALP, and AST after various treatments.

Furthermore, FeGA activation did not result in system damage, as shown in Figure 4. After the treatment, mice’s vital organs (heart, liver, spleen, lungs, and kidney) showed no inflammation and damage in the body, liver, and kidney indexes were also normal. Many nanomaterials have high therapeutic efficacy, but they also have a high risk of systemic toxicity, which limits their future clinical applications (Wang et al., 2019). The *in vivo* results show that our unique combined treatment not only achieves a high level of biological safety but also increases tumor •OH content and enhances the efficacy of RT with a powerful FH-enhanced therapy.

## Conclusion

In conclusion, by encapsulating FeGA nanoparticles in agarose hydrogel, we developed an injectable light-controlled hydrogel system as a FeGA reservoir and release controller FH. The nano-system can combine low-dose radiation with other treatments to improve tumor treatment outcomes. FeGA nanoparticles can be employed as an outstanding radiosensitizer and PTA due to the nanozyme and superior photothermal effect in the NIR-I region. The agarose hydrogel underwent regulated and reversible melting and softening states under the NIR laser power, resulting in light-triggered FeGA nanoparticles release and hydrogel deterioration. The release rate of FeGA nanoparticles can also be adjusted by changing the parameters. More importantly, by injecting the hydrogel intratumorally, the concentration of FeGA nanoparticles in tumor tissues will be significantly raised, allowing for one injection and multiple treatments *in vivo*. It is worth emphasizing that after photothermal treatment, we observed the mitochondrial damage of tumor cells, which considerably boosts radiotherapy efficacy. The FH exhibits outstanding cancer cell killing and tumor ablation properties in both *in vitro* and *in vivo* tests, with good stability, low toxicity, and biocompatibility. Thus, we may conclude that the FH has great anti-cancer potential in combination therapy.

## Data Availability

The original contributions presented in the study are included in the article/Supplementary Material, further inquiries can be directed to the corresponding authors.
