# Supplementary material for: Injectable Hydrogel for Synergetic Low Dose Radiotherapy, Chemodynamic Therapy and Photothermal Therapy
Source: Front Bioeng Biotechnol. 2021 Nov 22;9:757428. doi: 10.3389/fbioe.2021.757428 (PMC8645961; doi:10.3389/fbioe.2021.757428)
Supplement: Supplementary file 1 [file DataSheet1.docx]

Experimental Procedures

**Materials and reagents.**

Iron chloride hexahydrate (FeCl_3_·6H2O) and polyvinylpyrrolidone K30 (PVP) were purchased from Sinopharm Chemical Reagent Co., Ltd. Agarose was purchased from Yare Shanghai. Gallic acid were acquired from Macklin. MTT Cell Proliferation Assay Kit were obtained from Yeasen Biotech Co., Ltd (China). The other reagents used in this work were purchased from Sinopharm Chemical Reagent (China) and Aladdin-Reagent (China).

**Cell culture**

4T1 mouse breast cancer cell line was obtained from the Cell Bank of the Chinese Academy of Sciences and incubated in RPMI-1640 medium supplemented with 10% FBS in a humidified atmosphere at 37℃.

**Preparation and characterization of Fe-GA nanoparticles (FeGA)**

Fresh GA–Fe was prepared according to the literature.([1](#_ENREF_1)) FeCl3·6H2O was added into the gallate solution to keep a 1:1 stoichiometry of Fe^3+^ and GA^4−^ for reaction with magnetic stirring and nitrogen supplementation for 1 h, after which the suspension was centrifuged and the precipitate was washed, collected, and stored for further use. The morphology structures of FeGA were observed by the TEM (JEOL-2100). UV-vis spectra of different samples were recorded by the UV-vis spectrophotometry Lambda 35 (Perkin-Elmer). XPS spectra were recorded by ESCAlab250 (Thermal Scientific). Hydrodynamic diameter was detected by the dynamic light scattering (Nano-ZS ZEN3600).

**Preparation and characterization of FH**

The general protocol for the hydrogel preparation is as follows. The prepared FeGA (10 mg/mL in PBS) were mixed into 2% agarose solution to form FH. Wherein the concentration of FeGA was 200 μg/mL. Scanning electron microscopy (SEM) images were captured on a Hitachi FE-SEM S4800 instrument with an acceleration voltage of 3 kV.

**FeGA release study**

The in vitro FeGA release profile from FH was carried out. 1mL of FH containing 20 μg FeGA was added into culture dish. To investigate the stimuli effect of laser irradiation on the release behavior, the release experiment of FeGA was initially performed with or without 0.5 W/cm^2^ 808 nm laser irradiation. At appropriate time point, 100 μL of different samples were collected. The Fe content was quantitatively analyzed by ICP-MS.

**Rheological Test**

Rheology experiments were performed on an Anton Paar rheometer. Hydrogel samples of different temperatures were prepared and gently placed on the middle of a 15 mm diameter parallel plate with a proper gap. Dynamic oscillatory frequency sweep measurements were conducted at a 1% strain amplitude. To prevent the evaporation of water, a lid was prepared on the top.

**JC-1 fluorescence imaging**

For the JC-1 assay: 4T1 cells were incubated at 37 °C for 24 h in five groups: (1) PBS + NIR (808nm laser, 0.5W/cm^2^); (2) RT; (3) FH + NIR; (4) RT (6 Gy); (5) FH + NIR + RT. The FeGA concentration was 200 μg/mL in group 3 and 5. Firstly, the preheated FH (containing 400 μg FeGA) was dropped onto the center of the well plate (agarose 2%) and waited for solidification. Then the laser irradiation was carried out to make the FH dissolve and heat up. Then, cells in group 2, 4 and 5 were irradiated with the NIR. After 2 h, group 2, 4 and 5 were treated by RT. The dose of group 2 and 5 were 2 Gy. The dose of group 4 was 6 Gy. The cells were stained with JC-1 for 20 min before washing with PBS. Then, the mitochondrial damage/disruption was detected by fluorescence microscopy. In JC-1 experiments, For red fluorescence: Ex = 585 nm, Em = 590 nm, For green fluorescence: Ex = 514 nm, Em = 529 nm.

**γ-H_2_AX immunofluorescence analysis.**

4T1 cells were incubated at 37 °C for 24 h in five groups: (1) PBS + NIR (808nm laser, 0.5W/cm^2^); (2) RT; (3) FH + NIR; (4) RT (6 Gy); (5) FH + NIR + RT. The FeGA concentration was 200 μg/mL in group 3 and 5. Then, cells in group 2, 4 and 5 were irradiated with the NIR. After 2 h, group 2, 4 and 5 were treated by RT. The dose of group 2 and 5 were 2 Gy. The dose of group 4 was 6 Gy. After three-times rinse with PBS, cells were fixed in 4% paraformaldehyde for 20 min, treated by 0.1% Triton-X-100 and later blocked at room temperature for 2 h. Washed with PBS for three times, the cells were dyed with DAPI and secondary anti-γ H2AX antibody with 5% FBS and 1% Trition-X-100 before washed with PBS. The cells immunofluorescence was analyzed by a fluorescent microscope (IX81, Olympus, Japan).

***In vitro* ROS generation**

ROS generation was also assessed in vitro on 4T1 cells. Total hydroxyl radical detection was detected by intracellular hydroxyl radical detection kit, using confocal laser scanning microscope (CLSM) imaging. Briefly, 4T1 cells were incubated for 24 h with five different groups: (1) PBS + NIR (808nm laser, 0.5W/cm^2^); (2) RT; (3) FH + NIR; (4) RT (6 Gy); (5) FH + NIR + RT. The FeGA concentration was 200 μg/mL in group 3 and 5. Then, cells in group 2, 4 and 5 were irradiated with the NIR. After 2 h, group 2, 4 and 5 were treated by RT. The dose of group 2 and 5 were 2 Gy. The dose of group 4 was 6 Gy. All samples were added directly into the cells. Then, hydroxyl radical detection kit was used to detect different ROS level.

**Clonogenic survival assay**

500 cells per flask were seeded in 25 cm^2^ flasks and cultured for 24 h. Flasks were treated under following conditions: (1) PBS + NIR (808nm laser, 0.5W/cm^2^); (2) RT; (3) FH + NIR; (4) RT (6 Gy); (5) FH + NIR + RT. The FeGA concentration was 200 μg/mL in group 3 and 5. Then, cells in group 2, 4 and 5 were irradiated with the NIR. After 2 h, group 2, 4 and 5 were treated by RT. The dose of group 2 and 5 were 2 Gy. The dose of group 4 was 6 Gy. To allow formation of colonies, after irradiation, cells were washed and cultured in normal air for 11 days. To determine the clonogenic survival rate, cultures were first fixed with paraformaldehyde, and then stained with trypan blue. Colonies with greater than 50 cells were counted under the microscope, and the survival fractions (SF) were calculated using the formula SF = colonies counted/cells seeded.

**Biocompatibility of FeGA**

We then tested the biocompatibility of FeGA to other types of cells under dark condition. 4T1 cancer cells were seeded in 96-well plates at a density of 5 × 10^3^ cells per well and incubated for 24 h. Afterwards, cells were incubated for 6h with different concentrations of Fe^2+^ (0, 5, 10, 20 and 40 μg/mL). At the end of the incubation, 5 mg/mL MTT solution was added, and the plate was incubated for another 4 h. Finally, the absorbance values of the cells were determined by using a microplate reader (Emax Precision, USA) at 570 nm. The background absorbance of the well plate was measured and subtracted. The cytotoxicity was calculated by dividing the optical density (OD) values of treated groups (T) by the OD values of the control (C) (T/C × 100%).

**Animal tumor models**

Female BALB/c nude mice aged 4-5 week were purchased from Vital River Company (Beijing, China). 100 μL of 4T1 cell suspension (1×10^6^ cells per mL) were subcutaneous injected into each mouse to establish the tumor models. The animal experiments were carried out according to the protocol approved by the Ministry of Health in People’s Republic of PR China and were approved by the Administrative Committee on Animal Research of the Wuhan University.

***In vivo* infrared thermography**

To monitor the *in vivo* photothermal effect, FH (FeGA: 1 mg/kg) was intratumorally injected into the tumor-bearing mice, and then the tumors were irradiated by 0.5 W/cm^2^ laser irradiation for 10 min at 1 h post-injection. PBS injection used as control group. Meanwhile, the temperature at the tumor was monitored using an infrared camera (Fotric 225).

***In vivo* antitumor study**

The mice were firstly divided randomly into 6 groups (each group included 5 mice): 1) PBS + NIR group; 2) RT; 3) FH + NIR; 4) RT (6 Gy) 5) FH + NIR + RT. The injection method is intratumoral injection. Among them, the dose of FeGA in groups 3 and 5 are 1 mg/kg. NIR was conducted 1h after the injection. After 2 h, group 2, 4 and 5 were treated by RT. The dose of group 2 and 5 were 2 Gy. The dose of group 4 was 6 Gy. Mice body weight and tumor volume in all groups were monitored every 4 days. A caliper was employed to measure the tumor length and tumor width and the tumor volume was calculated according to following formula. Tumor volume = tumor length × tumor width^2^ / 2. After 16 days treatment, mice were sacrificed. Five main organs (heart, liver, spleen, lung and kidney) of all mice were harvested, washed with PBS, and fixed with paraformaldehyde for histology analysis. The blood samples from these mice (≈1 mL) were collected for blood biochemistry analysis. And the tumor tissues were weighed, and fixed in 4% neutral buffered formalin, processed routinely into paraffin, and sectioned at 4 μm. Then the sections were stained with hematoxylin and eosin (H&E) and finally examined by using an optical microscope (BX51, Olympus, Japan).

**Statistical analysis**

Data analyses were conducted using the GraphPad Prism 5.0 software. Significance between every two groups was calculated by the Student’s t-test. *P < 0.05, **P < 0.01, ***P < 0.005.


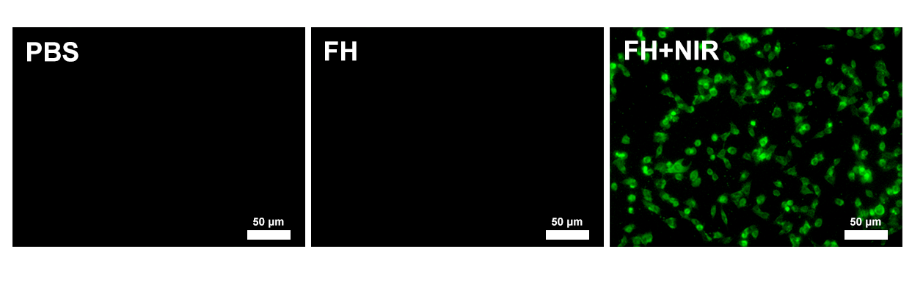


Figure S1. In vitro ·OH detection after different treatments.
